# Subclavian or axillary artery cannulation for extracorporeal membrane oxygenation: A systematic review

**DOI:** 10.1016/j.xjon.2025.101562

**Published:** 2025-12-18

**Authors:** Jan Coveliers, Paolo Meani, Mariusz Kowalewski, Eliza Huizinga, Karthik Gutta, Giulia Piccirillo, Emanuele Gasparotti, Emanuele Vignali, Marilena Mazzoli, Wouter Huberts, Hamed Moradi, Michele Di Mauro, Robert J. Holtackers, Monique de Jong, Sandro Gelsomino, Domenico Paparella, Simona Celi, Dorela Haxhiademi, Erik Körver, Arne Doddema, Michal J. Kawczynski, Samuel Heuts, Elham Bidar, Roberto Lorusso

**Affiliations:** a Cardio-Thoracic Surgery Department, Maastricht University Medical Centre (MUMC), Maastricht, The Netherlands; b Cardiovascular Research Institute Maastricht (CARIM), Maastricht, The Netherlands; c Cardiac Surgery Department, University Hospital Antwerp (UZA), Edegem, Belgium; d Faculty of Medicine and Health Sciences, University of Antwerp, Antwerp, Belgium; e Cardiothoracic and Vascular Anesthesia and Intensive Care Unit, ASST Grande Ospedale Metropolitano Niguarda, Milan, Italy; f Department of Cardiac Surgery, Central Clinical Hospital of the Ministry of Interior, Centre of Postgraduate Medical Education, Warsaw, Poland; g Thoracic Research Centre, Collegium Medicum, Nicolaus Copernicus University, Innovative Medical Forum, Bydgoszcz, Poland; h Cardiac Surgery Unit, University of Foggia, Italy; i BioCardioLab, Bioengineering Unit, Fondazione Monasterio, Massa, Italy; j Department of Biomedical Engineering, Eindhoven University of Technology, Eindhoven, The Netherlands; k Department of Medical and Surgical Sciences, University of Foggia, Division of Cardiac Surgery, Foggia, Italy; l Department of Radiology & Nuclear Medicine, Maastricht University Medical Centre (MUMC), Maastricht, The Netherlands; m Anesthesia and Intensive Care Unit, Fondazione Monasterio, Massa, Italy

**Keywords:** subclavian artery, axillary artery, ECMO, extracorporeal membrane oxygenation, cannulation, veno-arterial ECMO

## Abstract

**Background:**

Subclavian or axillary artery cannulation for venoarterial extracorporeal membrane oxygenation (VA-ECMO) provides a valuable alternative to femoral access; however, a comprehensive overview of such an approach in this setting is lacking. This review examined types of access, clinical complications, and outcomes of subclavian/axillary cannulation, emphasizing the pros and cons of this VA-ECMO approach as well as areas for further investigation.

**Methods:**

A systematic search was conducted in PubMed, Scopus, and Web of Science for articles published between January 2000 and December 2024. Studies were included that (1) reported on axillary or subclavian artery cannulation in VA-ECMO, (2) involved adult patients, and (3) provided in-hospital outcomes. Exclusion criteria were case reports with fewer than 5 patients and non-English language articles. Data on patient demographics, indications, cannulation techniques, complications, and in-hospital survival were collected.

**Results:**

Seventeen studies with a total of 2030 patients were selected and analyzed. Subclavian or axillary cannulation was predominantly right-sided (99%) and involved graft interposition in 81% of cases. Indications for VA-ECMO with this arterial access included acute myocardial infarction shock (11%-42%), acute decompensation with chronic heart failure (4.9%-52%), postcardiotomy shock (7%-75.2%), acute myocarditis (4%- 7.7%) and refractory respiratory distress (5%-50%). Complications included limb ischemia (1.2%-9.6%), stroke (5.2%-18.8%) and bleeding events (4%-37.5%, often at the graft site). Left ventricular (LV) unloading strategies were used in 5.2% to 15.0% of cases but were not documented in almost 60% of the studies. In-hospital and 1-year survival rates ranged from 82.7% to 55% and from 50.6% to 25.7%, respectively.

**Conclusions:**

VA-ECMO with axillary or subclavian artery access can be a safe and effective configuration, particularly for patients with contraindications or need of change of femoral cannulation. However, conflicting data on stroke risk, hemodynamic effects on LV ejection, the need for LV unloading (particularly with the right-sided approach), and both short- and long-term survival rates warrant further investigation.

**Figure:**
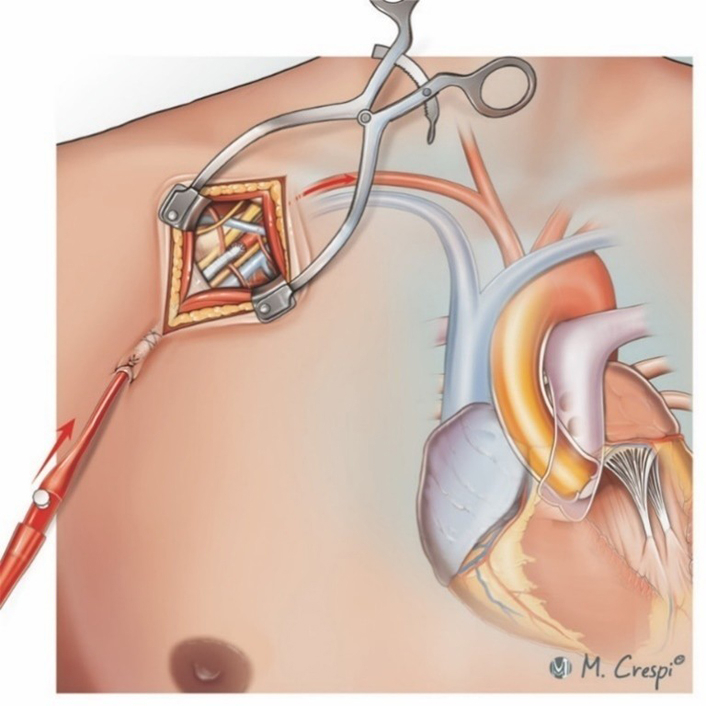
Subclavian/axillary cannulation for VA-ECMO using an interposition graft with the cannula tip in the graft.

**Central Message**

Axillary or subclavian artery cannulation for VA-ECMO offers antegrade flow and less limb ischemia than femoral access but carries higher risks of stroke and bleeding, requiring tailored management.

**Perspective**

Axillary or subclavian cannulation enables antegrade perfusion and improved cerebral oxygenation during VA-ECMO, reducing limb ischemia compared with femoral access. However, its technical complexity, bleeding, and stroke risk highlight the need for refined techniques, standardized protocols, and focused research on hemodynamic and neurologic outcomes.

Venoarterial extracorporeal membrane oxygenation (VA-ECMO) is a life-saving intervention for patients with refractory cardio-circulatory-respiratory compromise.^1^^,^^2^ The choice of arterial cannulation site may have a significant influence on VA-ECMO management, complications, and overall success/outcomes of the procedure.^3^^,^^4^ While femoral artery cannulation remains the most common access and is considered the standard in most cases, alternative sites such as the subclavian or axillary arteries are gaining attention for their potential advantages, particularly in specific patient populations.^5^^,^^6^

The published reports of VA-ECMO with subclavian or axillary artery cannulation are mainly single-center series and frequently compare axillary cannulation with central or femoral cannulation.^7^ As a result, the interpretation of VA-ECMO experiences with subclavian or axillary artery cannulation in terms of prevalence, patient selection, in-hospital management, and short- and long-term outcomes, are difficult owing to the limited available information.

The present study aimed to provide a detailed and comprehensive evaluation of published series of patients undergoing VA-ECMO with perfusion access at the axillary or subclavian artery. In addition, several reports with limited patient cohorts comparing different arterial access routes for VA-ECMO in adult patients, as well as gaps in knowledge and areas warranting further investigations in this setting, were assessed.

## Methods

This systematic review was performed according to the PRISMA statement and is registered at PROSPERO (CRD420250643971). Ethical approval and informed consent were not required for this systematic review, as it was based exclusively on previously published data.

### Eligibility Criteria

This review included a range of study designs to evaluate outcomes and complications of subclavian/axillary artery cannulation for VA-ECMO support. Eligible designs included observational studies, such as prospective and retrospective cohort studies, and case-control studies comparing subclavian or axillary cannulation to alternative arterial access methods like femoral or central aorta cannulation. Comparative studies analyzing differences in complications, survival outcomes, or procedural risks were included, along with case series of 5 or more patients focusing on subclavian/axillary artery cannulation. Excluded publications were case reports involving fewer than 5 patients, studies lacking detailed outcome data, non–VA-ECMO studies (eg, aortic surgery), non-English language articles, and pediatric studies involving patients age <18 years. In the event of multiple publications using the same database, the study with the longest patient-year follow-up and most complete data was included.

### Information Sources

A systematic search was conducted in the PubMed, Embase, and Web of Science databases from January 2000 to December 2024. The search was last performed on January 31, 2025. No additional grey literature databases or clinical trial registries beyond PubMed, Embase, and Web of Science were systematically queried.

### Search Strategy

The full search strategies for each database—including keyword terms, Boolean operators, and applied filters—are detailed in Table E4. These strategies were reviewed collaboratively by the study authors and validated by an independent academic librarian to ensure transparency and reproducibility.

### Selection Process

Two independent reviewers (JC, PM) screened abstracts and then full texts of studies. Disagreement was resolved by discussion with the senior author (RL).

### Data Collection Process

Data were extracted to SPSS Statistics for Windows version 28.0 (IBM) by independent reviewers, who subsequently checked one another's entries and data integrity. Although kappa statistics were not formally calculated, interrater agreement was considered high following pilot calibration of the selection criteria.

### Data Abstraction/Synthesis

Data extraction was focused on (1) baseline patient characteristics, including demographics, clinical conditions, and indications for VA-ECMO; (2) cannulation characteristics, including site of cannulation (subclavian or axillary), cannulation technique (direct cannulation, side graft interposition, or percutaneous access), laterality, and method of limb protection; and (3) clinical outcomes, such as survival rates (eg, 30 days, 1 year), VA-ECMO weaning success, and complications (eg, limb ischemia, stroke, bleeding). Complications were defined based on explicit mention in the included studies and typically included vascular access complications (eg, bleeding, limb ischemia, stroke, infection, hyperperfusion syndrome). Survival outcomes were extracted as reported, with in-hospital or 30-day mortality and 1-year survival rates provided most frequently. Whenever possible, standardized definitions were cross-verified with study methodology or appendices; however, variability across studies in complication definitions was accepted as a reflection of real-world practice. Studies reporting outcome comparisons between different access routes were included as well.

The data synthesis for this review used both qualitative and quantitative methods. Meta-analyses were conducted using the inverse variance method for pooling effect estimates. Between-study heterogeneity was quantified using the DerSimonian-Laird estimator to calculate τ^2^ values. Proportions were transformed using the logit scale to stabilize variances prior to pooling. For individual studies, 95% confidence intervals were computed using the Clopper-Pearson exact method to ensure accurate coverage, particularly for proportions near 0 or 1. Given the limited number of studies per outcome and substantial clinical heterogeneity in indications, patient populations, and definitions, formal sensitivity or subgroup analyses were not conducted. Future meta-analyses with more granular and standardized data may enable such comparisons.

Venous cannulation strategies were described in various ways. Most studies did not systematically report the site or technique of venous access. In those that did, femoral venous cannulation was commonly used, with percutaneous access preferred in urgent scenarios. Owing to this heterogeneity, a comparative analysis of venous approaches was not feasible.

Data abstraction focused on survival outcomes (eg, 30-day and 1-year survival), VA-ECMO weaning success, and complication rates as reported in each study. No retrospective reclassification of outcomes or definitions was done when inconsistencies were present, which is acknowledged as a potential source of heterogeneity. Extracted data were summarized descriptively to highlight procedural techniques, patient selection criteria, hemodynamic implications, and complications specific to subclavian or axillary cannulation. This mixed-methods approach enabled a comprehensive evaluation of the clinical utility and challenges of this arterial access strategy.

## Results

**Figure 1:**
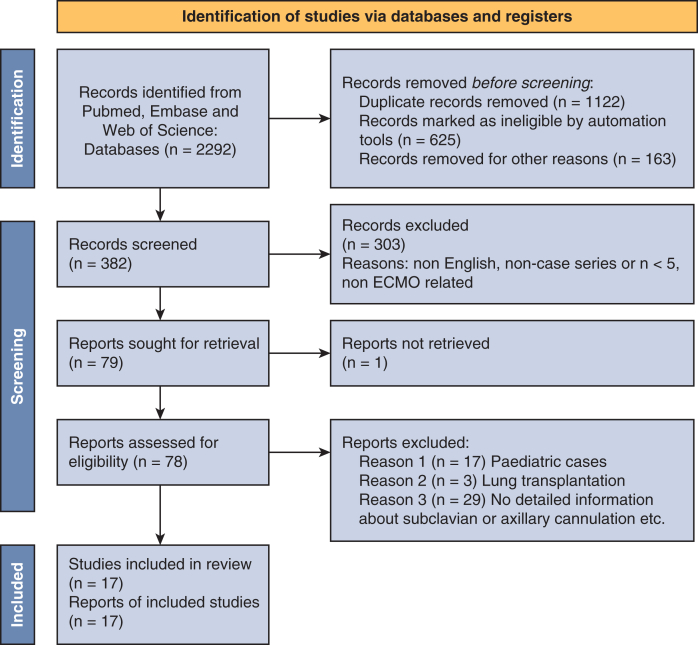
PRISMA flow chart of the search strategy.

The literature search yielded in 17 studies published between 2000 and 2024 that met the inclusion criteria. The PRISMA flow chart of the search strategy is shown in Figure 1. Sample sizes ranged from 5 to 397 patients. The 17 studies included a total of 2030 patients receiving VA-ECMO via axillary or subclavian artery cannulation.

### Baseline Patient and VA-ECMO Characteristics

Baseline characteristics, shock etiologies, and outcomes for the study patients undergoing VA-ECMO with subclavian or axillary cannulation are summarized in Table 1. Postcardiotomy shock (51.44%), acute cardiac decompensation with chronic heart failure (23.79%), and acute myocardial infarction (AMI; 17.27%) were the most frequently reported clinical scenarios leading to VA-ECMO initiation.^4^^,^^5^^,^^8^, ^9^, ^10^, ^22^, ^11^, ^23^, ^12^, ^13^, ^14^, ^15^, ^16^, ^17^, ^18^, ^19^, ^20^, ^21^

**Table 1:** Baseline characteristics of patients with subclavian or axillary cannulation for VA-ECMO

Study details				Etiology, %				
Year	First author	Period	Cases, n	Acute on chronic	Post- cardiotomy	AMI	Myocarditis	Respiratory
2003	Moazami^8^	N/A	5	N/A	60	20	N/A	N/A
2005	Navia^9^	2001-2003	5	N/A	60	N/A	N/A	40
2012	Javidfar^10^	2009-2011	20	25	50	20	5	50
2013	Chamogeorgakis^4^	2001-2011	81	4.90	61.70	16.00	N/A	13.60
2017	Ranney^22^	2009-2015	16	25.00	6.30	12.50	N/A	12.50
2017	Wong^11^	2010-2015	41	17.0	7.0	37.0	N/A	5.0
2017	Hysi^23^	2004-2016	246	N/A	N/A	N/A	N/A	N/A
2021	Radakovic^12^	2010-2019	19	N/A	N/A	N/A	N/A	N/A
2021	Pisani^13^	2013-2017	174	13.80	55.2	17.80	4.0	N/A
2020	Ohira^14^	2009-2019	218	25.7	30.3	27.1	N/A	N/A
2022	Moussa^15^	2013-2019	52	23.10	26.9	44.2	7.7	N/A
2022	Schaefer^16^	2000-2019	250	N/A	75.2	N/A	N/A	N/A
2023	Radwan^5^	2014-2019	179	N/A	N/A	N/A	N/A	N/A
2024	Chiarini^17^	2013-2019	397	N/A	52.0	32.5	N/A	6.40
2025	Do Vale^18^	2013-2019	209	26.0	32.0	11.0	N/A	N/A
2024	Jin^19^	2019-2022	51	N/A	N/A	N/A	N/A	N/A
2024	Nersesian^20^	2020-2022	67	52	24	19.5	4.5	N/A
Total cases, mean (95% CI)	2030	22.2 (15.4-31.0); *I*^2^ = 83.8; *P* < 01	35.1 (23.5-48.9); *I*^2^ = 94.6; *P* < .001	23.1 (17.3-30.1); *I*^2^ = 81.9; *P* < .001	5.0 (3.0-8.0); *I*^2^ = 0; *P* = .761	15.5 (6.4-33.0); *I*^2^ = 86.4; *P* < .001		

*I*^2^ denotes the Higgins test percentage for heterogeneity, and the *P* value indicates whether this heterogeneity is statistically significant.

*AMI*, Acute myocardial infarction; *N/A*, not available; *CI*, confidence interval.

Details of access techniques and cannulation-related complications are provided in Table 2. Axillary cannulation was reported in 92% of cases, the right side was chosen in 99% of cases,^4^^,^^5^^,^^8^, ^9^, ^10^, ^11^^,^^23^, ^12^, ^13^, ^14^, ^15^, ^16^, ^17^, ^18^, ^19^, ^20^^,^^22^ and graft interposition was present in 81% of cases.^4^^,^^5^^,^^8^, ^9^, ^10^, ^22^, ^11^, ^23^, ^12^, ^13^, ^14^, ^15^, ^16^, ^17^, ^18^, ^19^, ^20^, ^21^ Gelsoft and Hemashield grafts were the most frequently used graft types.^4^^,^^5^^,^^8^, ^9^, ^10^, ^22^, ^11^, ^23^, ^12^, ^13^, ^14^, ^15^, ^16^, ^17^, ^18^, ^19^, ^20^, ^21^ The use of cannulated limb protection measures for limb ischemia or hyperperfusion was limited. In some cases, external measures, such as elastic bandages or invasive bilateral monitoring, were applied,^14^ while in others, the use of invasive techniques, such as restrictive vessel-loops^11^^,^^16^ and distal perfusion catheters, on indication^5^^,^^24^ or systematically, were instituted.^13^ Restrictive vessel loops were applied as a temporary measure to modulate arterial flow in cases of suspected or confirmed hyperperfusion syndrome of the upper limb. This technique was used most often in graft-based cannulation when excessive flow was directed toward the ipsilateral extremity.

**Table 2:** Cannulation techniques for subclavian and axillary cannulation in VA-ECMO

First author	Axillary or subclavian cannulation (% of the total VA-ECMO population)	Direct or graft-based cannulation	Type of graft	Laterality	Limb protection
Moazami^8^	Axillary	Graft-based	Gelsoft plus	Right	N/A
Navia^9^	Axillary	Graft-based (80%)	Hemashield	N/A	N/A
Javidfar^10^	Subclavian	Graft-based	Gelweave	Right	N/A
Chamogeorgakis^4^	Axillary (26.3%)	Graft-based	Hemashield	Right	N/A
Ranney^22^	Axillary (12.2%)	Graft-based	Dacron	Right	N/A
Wong^11^	Axillary (40.0%)	Graft-based	Hemashield	Right	Restrictive vessel-loop
Hysi^23^	Axillary	Direct	N/A	Right (90.6%)	N/A
Radakovic^12^	Axillary (N/A)	Graft-based	Dacron	Right	N/A
Pisani^13^	Axillary	Direct	N/A	Right	DPC (systematic)
Ohira^14^	Axillary (58.7%)	Graft-based	Hemashield	Right (95.4%)	Elastic bandage
Moussa^15^	Subclavian and axillary (13.9%)	Graft-based (N/A) Direct (N/A)	Dacron	Right	N/A
Schaefer^16^	Axillary (57.3%)	Graft-based	Dacron	Right	Restrictive vessel-loop
Radwan^5^	Axillary	Direct	N/A	N/A	DPC
Chiarini^17^	Axillary (20.9%)	Graft-based (N/A) Direct (N/A)	N/A	N/A	N/A
Do Vale^18^	Axillary (62.8%)	Direct	N/A	Right	DPC
Jin^19^	Combined axillary-femoral (47%)	Graft-based	Dacron	N/A	N/A
Nersesian^20^	Axillary (100%)	Graft-based (bifurcated)	GELSOFT Vascutek AX Bifem	Right	N/A

*VA-ECMO*, Venoarterial extracorporeal membrane oxygenation; *N/A*, not available; *DPC*, distal perfusion cannula.

### VA-ECMO Management and Hemodynamics

The time from presentation to cannulation was reported inconsistently. Postcardiotomy patients typically were cannulated intraoperatively or immediately postoperatively, while ST-elevation AMI and acute heart failure cohorts had more variable intervals, reflecting interhospital transfers and stabilization periods. Details on VA-ECMO runs and LV unloading are provided in Table 3. VA-ECMO duration was typically between 3 and 7 days, although some authors reported durations as short as 1 day and as long as 30 days.^20^ VA-ECMO duration stratified by etiology was not systematically reported across the studies. While cross-referencing run times with predominant cohort etiologies might suggest shorter durations in postcardiotomy patients and longer runs in heart failure patients, this approach risks overinterpretation. ECMO flow rates, when provided, averaged 3.84 L/minute (Table 3).

**Table 3:** Characteristics of support for patients with subclavian or axillary cannulation for VA-ECMO

First author	No. of cases	VA-ECMO flow, L/min	VA-ECMO duration, d∗	LV unloading, %†	LV unloading strategy, %				
					LV venting‡	Impella CP/5.0/5.5	Atriosept-ostomy	LV apex	IABP
Moazami^8^	5	3.5-5	3 (2-7)	N/A	N/A	N/A	N/A	N/A	N/A
Navia^9^	5	N/A	3-20	N/A	N/A	N/A	N/A	N/A	N/A
Javidfar^20^	20	4.24	7 (1-30)	N/A	N/A	N/A	N/A	N/A	N/A
Chamogeorgakis^4^	308	N/A	3.53 ± 0.86	N/A	N/A	N/A	N/A	N/A	N/A
Ranney^22^	131	N/A	4 (3-7)	N/A	N/A	N/A	N/A	N/A	N/A
Wong^11^	103	N/A	5 (2-10)	17.0	17.0	N/A	N/A	N/A	N/A
Hysi^23^	246	N/A	6.5 ± 4.7	N/A	N/A	N/A	N/A	N/A	N/A
Radakovic^12^	158	3.45 ± 0.84	N/A	13.6	N/A	11.4	N/A	2.3	N/A
Pisani^13^	174	N/A	7 (1-26)	5.20	N/A	N/A	5.20	N/A	N/A
Ohira^14^	371	N/A	6	N/A	N/A	11.8	N/A	N/A	47.7
Moussa^15^	372	1.5-5.5	14 (8 to not observed)	17.3	3.80	13.50	0.50	1.9	1.9
Schaefer^16^	436	N/A	4.64 (2.91-7.09)	N/A	N/A	N/A	N/A	N/A	N/A
Radwan^5^	179	N/A	8.4 ± 5.1	N/A	N/A	N/A	N/A	N/A	N/A
Chiarini^17^	1897	N/A	5 (3-8)	15.0	N/A	N/A	N/A	N/A	N/A
Do Vale^18^	534	N/A	6 (4-8)	13	N/A	N/A	9.0	N/A	N/A
Jin^19^	24	N/A	8.45 ± 7.63	N/A	N/A	N/A	7.0	N/A	N/A

*VA-ECMO*, Venoarterial extracorporeal membrane oxygenation; *LV*, left ventricular; *IABP*, intra-aortic balloon pump; *N/A*, not available.

∗ Data expressed as mean ± standard deviation or median (interquartile range).

† Patient might have more than 1 LV unloading strategy, escalation of devices, or undefined overall management.

‡ Undefined LV unloading strategy.

When described, the reported rate of LV unloading was between 5.2% and 15.0% of cases, but it was not documented in almost 60% of the studies. Only 1 study reported LV unloading details, with Impella used in 13.5% of cases, atrioseptostomy in 0.5%, and LV venting in 3.80%.^12^

Anticoagulation protocols were described inconsistently across the included studies. While some studies referenced standard VA-ECMO anticoagulation strategies (eg, unfractionated heparin targeting activated clotting time or activated partial thromboplastin time ranges), most did not provide details on dosing, monitoring parameters, or adjustments made during graft-based cannulation, limiting comparative interpretation of bleeding and thrombotic outcomes. Data on revascularization procedures (percutaneous coronary intervention or coronary artery bypass grafting) following VA-ECMO initiation and the timing of such interventions were not systematically reported across studies. This is an important area for future research, particularly in AMI cohorts, in whom optimal timing may impact outcomes.

### Complications and Outcomes

Complications and outcomes are summarized in Table 4. Bleeding complications at cannulation sites varied between 4% and 37.5%, with higher rates of bleeding seen with graft-based cannulation techniques. Vascular complications included limb ischemia (1.2%-9.6%) and cerebrovascular events, with stroke rates ranging from 5.2% to 18.8%. Specific reporting of thromboembolic events and transient ischemic attacks was limited, precluding precise incidence determination. Infection rates were between 0% and 9%. Hyperperfusion syndrome was described as excessive perfusion of the ipsilateral limb, presenting with edema, pain, and, in rare cases, compartment syndrome requiring fasciotomy. It occurred more often with graft-based techniques, with rates as high as 24.70% of cases. Severe sequelae such as limb loss or neurologic impairment were not reported systematically. The incidence of pulmonary edema, when reported, varied from 1.3% to 28.8%. Rates of successful VA-ECMO weaning varied between 30.9% and 80%.^4^^,^^5^^,^^8^, ^9^, ^10^, ^11^^,^^23^, ^12^, ^13^, ^14^, ^15^, ^16^, ^17^, ^18^, ^19^, ^20^^,^^22^ In-hospital or 30-day survival ranged from 17.3% to 63.8%, and 1-year survival rates ranged from 25.7% to 50.6% (Table 4).

**Table 4:** Complications and outcomes of patients with subclavian or axillary cannulations for VA-ECMO

Study details		Complications, %							Outcomes, %		
Year	First author	Bleeding	Infection	Limb hyperperfusion syndrome	Brachial plexus injury	Limb ischemia	Stroke rate	Pulmonary edema	Successful VA- ECMO weaning	In-hospital or 30-d survival	1-y survival
2003	Moazami^8^	N/A	N/A	N/A	N/A	N/A	N/A	N/A	40.00	40.00	N/A
2005	Navia^9^	20	N/A	N/A	N/A	N/A	N/A	N/A	80.00	40.00	N/A
2012	Javidfar^20^	5	5	5	N/A	N/A	N/A	N/A	50.00	45.00	N/A
2013	Chamogeorgakis^4^	17.30	0	24.70	2.50	1.20	N/A	1.3	30.9	17.30	N/A
2017	Ranney^22^	37.50	0	N/A	N/A	6.30	18.8	N/A	N/A	56.20	N/A
2017	Wong^11^	22	N/A	15	N/A	4.80	7.3	N/A	66.00	37.0	N/A
2017	Hysi^23^	4.50	N/A	N/A	1.63	0	N/A	N/A	N/A	N/A	N/A
2021	Radakovic^12^	N/A	N/A	N/A	N/A	N/A	N/A	N/A	47.7	22.7	N/A
2021	Pisani^13^	4	1.70	N/A	0.6	1.1	9.20	1.70	51.1	63.8	50.6
2021	Ohira^14^	15.1	2.8	2.30	N/A	0	12.4	N/A	78.4	60.6	N/A
2022	Moussa^15^	26.9	N/A	N/A	N/A	9.6	N/A	28.8	34.6	57.7	38.5
2022	Schaefer^16^	5.2	4.0	N/A	N/A	4.8	11.2	N/A	58.4	48.8	N/A
2023	Radwan^5^	13.4	N/A	N/A	N/A	6.1	6.15	N/A	48.6	34.6	25.7
2024	Chiarini^17^	13.8	N/A	N/A	N/A	3.80	15.9	N/A	52.8	54.7	43.6
2024	Do Vale^18^	N/A	N/A	N/A	N/A	4.8	5.2	13.0	71.3	55.5	N/A
2024	Jin^19^	14	N/A	N/A	N/A	4.17	8.33	N/A	62.5	58.3	N/A
2024	Nersesian^20^	13	9	N/A	N/A	4.5	N/A	N/A	58	48	N/A

*VA-ECMO*, Venoarterial extracorporeal membrane oxygenation; *N/A*, not available.

### Comparative Studies

Comparative studies are depicted in Table E1. Studies comparing axillary artery cannulation with femoral artery cannulation reported comparable 90-day mortality (54% for axillary cannulation and 58% for femoral cannulation).^14^, ^15^, ^16^^,^^18^ Axillary cannulation was associated with higher rates of limb ischemia, local infections, and wound complications (5%, 3%, and 3% respectively) but a higher stroke rate (11%, vs 2% with femoral artery cannulation). Studies comparing central (aortic), subclavian/axillary, and femoral cannulation^4^^,^^11^^,^^17^^,^^22^ reported that approximately one-third of VA-ECMO patients experienced cannulation-related complications, with complication rates of 34% for axillary cannulation, 36% for femoral cannulation, and 23% for central cannulation (Table E1). The rate of bleeding was higher in axillary cannulations (17.3%, vs 5.4% with femoral cannulation and 1.6% with central cannulation). Limb ischemia was more frequent with femoral cannulation (16.3% vs 1.2% with axillary cannulation and 4.9% with central cannulation; *P* < .01). The rate of hyperperfusion syndrome was higher in axillary cannulation (24.7% vs 4.9% in femoral cannulation and 1.6% in central cannulation; *P* < .01).

In a multicenter study of 1897 patients,^17^ major neurologic complications were documented in 19.6% of patients with axillary/subclavian cannulation, 15.8% of those with central aortic cannulation, and 11.9% of those with femoral cannulation (*P* < .001), while seizures occurred in 3.4%, 1.8%, and 1.3% of patients, respectively. A single-center study of 131 VA-ECMO patients found a 31.1% rate of axillary artery complications, a 9.1% rate of femoral artery complications, and an 11.1% rate of chest exploration for mediastinal bleeding with central cannulation. There were no significant differences in stroke rates or survival among the 3 groups.^22^ Among 81 patients undergoing axillary side-graft cannulation, 24.7% developed hyperperfusion syndrome and 17.3% experienced bleeding complications, whereas femoral cannulation was associated with a 16% rate of lower limb ischemia and a 10.8% rate of fasciotomy. In-hospital survival did not differ significantly across the cannulation methods (Table E1).

## Discussion

This systematic review provides, to the best of our knowledge, the first comprehensive review of the pre-ECMO patient characteristics, cannulation techniques, complications, and clinical outcomes associated with subclavian artery and axillary artery cannulation in patients undergoing VA-ECMO, either as an isolated approach or compared to alternative access routes such as the femoral artery or central aorta. The findings demonstrate that axillary or subclavian arterial access sites offer several potential benefits compared to femoral artery cannulation, particularly in terms of reduced risk of limb ischemia and greater patient mobility. However, the review also highlights important complications, such as hyperperfusion syndrome and a higher rate of acute brain injury; variability in outcomes across studies; and the still-unclear effects of such an approach in terms of the impact on LV hemodynamics, underscoring the need for further investigations and research in these settings.

### Patient Characteristics

The studies included in this systematic review highlighted various clinical indications, often involving patients with severe cardiac or respiratory failure who required prolonged or high-flow VA-ECMO support. Subclavian or axillary arteries are a valuable alternative for arterial access in VA-ECMO when femoral or central cannulation is contraindicated or less suitable. The main clinical indications favoring axillary or subclavian cannulation are summarized in Table E2. These routes were particularly indicated in patients with severe peripheral vascular disease,^4^^,^^9^ groin infection,^4^^,^^14^ or prior surgery that compromised femoral access. They are usually considered advantageous for providing antegrade arterial flow^4^^,^^16^ and ensuring better oxygenation of the brain and heart, making it ideal for addressing or preventing differential hypoxemia (harlequin syndrome), as well as favoring cerebral and coronary perfusion. Subclavian or axillary cannulation is especially beneficial for patients requiring prolonged VA-ECMO support,^13^^,^^15^ as it allows easier patient mobility^13^^,^^15^ and reduces the risks of limb ischemia^16^^,^^18^ as well as wound complications, particularly infection, thereby improving both short- and long-term outcomes.^4^^,^^10^^,^^13^^,^^14^^,^^22^

### Cannulation Techniques

An overview of cannulation techniques, including direct, graft-based, percutaneous, and bifurcated graft strategies, is provided in Table E3. This review found that 99% of subclavian or axillary cannulations were performed at the right side, likely owing to usual surgical access considerations during cardiac surgery (particularly aortic or redo surgery). The prevalence of preexisting devices such as automated implantable converter-defibrillators, defibrillators, or pacemakers was not systematically reported across studies, limiting assessment of their influence on cannulation laterality. Future studies should address this aspect, which may impact surgical planning and side selection. The choice between subclavian and axillary access depends on institutional protocols/policy and patient-specific anatomy. This review found that the axillary artery is favored for its ease of exposure and reduced risk of brachial plexus injury compared to the subclavian artery, which lies deeper and is more challenging to access without thoracic involvement.^25^ The subclavian artery is larger, however, which may favor the insertion of larger cannula in the event of need or preference.

**Figure 2:**
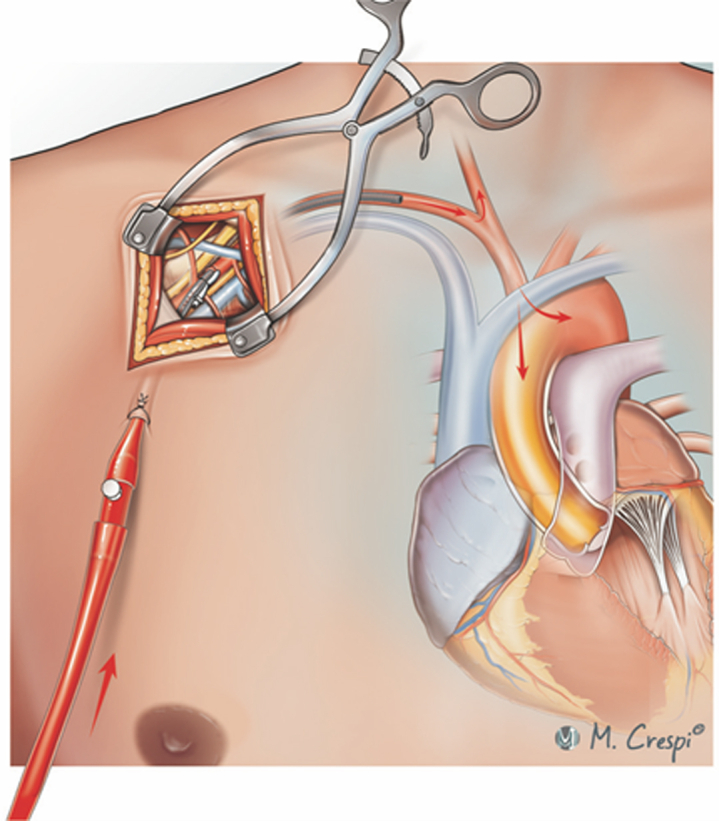
Techniques for subclavian/axillary artery cannulation in venoarterial extracorporeal membrane oxygenation: surgical cutdown and direct cannulation of the target artery after purse-string suture and tourniquet fixation. The *red arrows* indicate the direction of arterial blood flow following axillary/subclavian artery cannulation.

**Figure 3:**
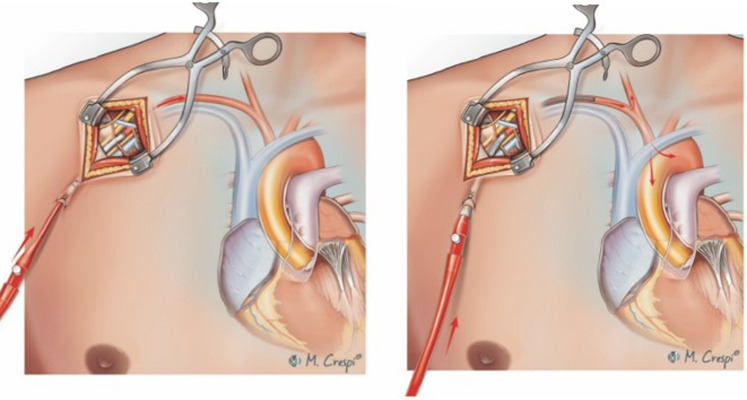
Techniques for subclavian/axillary artery cannulation in venoarterial extracorporeal membrane oxygenation: cannulation with interposition graft with cannula tip in the graft (A) or in the artery (B). The *red arrows* indicate the direction of arterial blood flow following axillary/subclavian artery cannulation.

Subclavian or axillary artery cannulation for VA-ECMO can be performed using several techniques, each tailored to the patient's clinical needs, urgency of VA-ECMO initiation, anticipated duration of circulatory assistance, support modality as well as configuration, and, finally, center-based expertise and policies. Direct cannulation involves surgical access and insertion of the cannula into the artery without any graft interposition (Figure 2), making it simpler and faster but less suitable for prolonged VA-ECMO owing to potential arterial wall damage and a higher risk of dislocation or vascular injury.^5^^,^^9^^,^^13^^,^^15^^,^^17^^,^^18^^,^^21^^,^^23^^,^^24^ The end-to-side graft interposition technique is gaining in popularity and uses a Dacron or PTFE graft sewn onto the subclavian or axillary artery to preserve the native vessel's integrity (Figure 3), reduce arterial damage, ensure prolonged support, and enhance patient mobility.^4^^,^^8^, ^9^, ^10^, ^11^, ^22^, ^12^^,^^14^^,^^17^^,^^19^,^22^

Tunneling of cannulas was not performed universally and was reported as a center-specific practice to reduce infection risk and enhance cannula stability.

The choice of graft size for axillary or subclavian cannulation was reported inconsistently across the included studies. When described, Dacron or PTFE grafts ranging from 6 to 10 mm in diameter were used, with larger sizes favored for higher anticipated VA-ECMO flows (>4.0 L/minute). However, specific criteria correlating graft size with vessel caliber or flow targets were seldom standardized. This lack of uniformity underscores the need for tailored graft selection based on patient-specific anatomy, flow requirements, and duration of support. The end-to-side graft interposition technique is more time-consuming and carries such risks as bleeding, infection, and limb hyperperfusion syndrome, especially when the cannula tip is positioned outside the artery along the graft, that is, distal to the end-to-side anastomosis. Some authors advocate advancing the cannula through the graft with the tip positioned inside the artery (Figure 3), to reduce the risk of bleeding at the anastomotic site due to the flow-related pressure exerted by the perfusion cannula.^9^^,^^10^ This nuance is reported inconsistently and might impact limb ischemia and hyperperfusion rates.^26^

Percutaneous cannulation is a minimally invasive option offering rapid access using ultrasound or fluoroscopic guidance, which is ideal for emergencies but less durable and associated with a higher risk of arterial injury, such as dissection or pseudoaneurysm formation, particularly after decannulation. The hybrid approach combines percutaneous access followed by a surgical stabilization of the cannula, providing a balance of efficiency and durability, making it suitable for situations requiring rapid initiation and longer-term support.^5^^,^^18^^,^^23^^,^^24^

**Figure 4:**
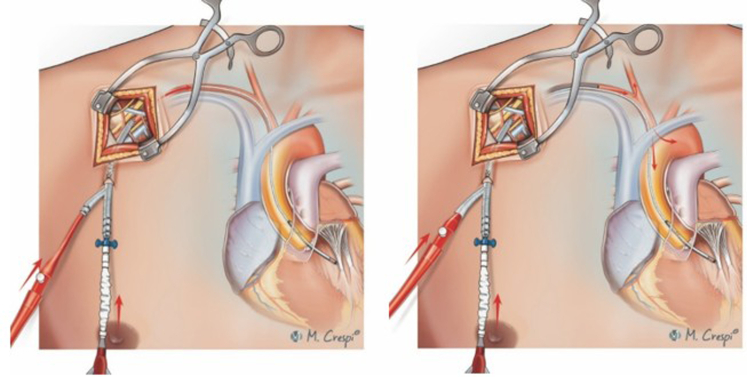
Techniques for subclavian/axillary artery cannulation in venoarterial extracorporeal membrane oxygenation (VA-ECMO). Cannulation with bifurcated graft with the cannula tip in the graft (A) and the cannula tip in the artery (B) for combined use of VA-ECMO with an Impella for left ventricular unloading. The *red arrows* indicate the direction of arterial blood flow following axillary/subclavian artery cannulation.

Recently a new approach has been described combining VA-ECMO with the Impella LV assist device through a single arterial access technique using a bifurcated graft on the axillary artery with one graft arm for the VA-ECMO–related perfusion cannula and the second arm for Impella device implantation (Figure 4).^20^ This approach represents an attractive access configuration for severe cardiogenic shock with high-flow circulatory support and simultaneous LV unloading.^20^ The goal of this technique is to reduce access sites and related complications, making a bedside-staged weaning (first VA-ECMO cannula followed by Impella removal) and final graft resection possible.^20^ This technique is still not widely adopted, however, and experience is limited.

A recent article highlighted the benefits of combining femoral and axillary artery cannulation in VA-ECMO patients compared to femoral artery cannulation alone.^19^ Despite the higher preoperative risk in the combined femoral and axillary artery group, this approach demonstrated improved hemodynamic stability, reduced limb ischemia, and a lower incidence of north-south syndrome. The combined femoral and axillary artery strategy offered superior systemic blood distribution and organ perfusion, as evidenced by better lactate levels and post–VA-ECMO laboratory value improvements.^19^ The dual-cannulation strategy is particularly advantageous for patients with severe cardiac dysfunction, enhancing perfusion to both the upper body and lower body.^19^ These findings underscore the importance of tailoring VA-ECMO strategies to individual patient conditions as well as hemodynamic needs, and suggest further research is needed to confirm these results or optimal VA-ECMO–based configurations in larger studies.

To summarize, each technique has specific advantages and limitations, with direct cannulation suited for short-term VA-ECMO, side graft interposition preferred for patients requiring prolonged support, and percutaneous or hybrid methods reserved for emergencies or high-risk scenarios. The choice of technique should consider the patient's anatomy, clinical condition, support duration and recovery or need for more advanced therapy transition, and surgical expertise and resources available.

### Hemodynamic Considerations

When using the subclavian or axillary artery in VA-ECMO, careful attention to hemodynamic effects, or VA-ECMO flow–LV ejection fraction interplay, is essential to optimize outcomes and minimize complications. Most articles describe flow from the axillary artery as an antegrade arterial flow,^4^^,^^9^^,^^14^ providing, as mentioned earlier, antegrade systemic and cerebral perfusion and reducing the risk of differential hypoxemia, which is common with retrograde femoral flow.^27^ This antegrade flow supports better oxygen delivery to the brain and heart while minimizing mixing in the aortic arch.^28^ Flow direction in VA-ECMO with subclavian or axillary artery cannulation is more complex, however.^29^ In certain anatomic configurations, subclavian or axillary cannulation in VA-ECMO can result in higher blood flow toward the aortic valve in the ascending aorta, depending on the location of the brachiocephalic artery entry site and alignment toward the aortic arch. Indeed, the flow direction—particularly from the right axillary or subclavian artery—might proceed preferably toward the aortic valve in a retrograde fashion and more forcefully than a more distal femoral artery–based retrograde flow in cases of a vertical and proximal ascending aorta vascular entry site of the brachiocephalic branch.^28^^,^^30^ This unfavorable anatomic pattern can increase LV afterload, potentially causing reduced or absent aortic valve opening during systole, along with LV distention, blood stasis, thrombus formation,^31^ and pulmonary congestion and edema.^31^ This complication is a significant concern and may require such interventions as atrial septostomy, LV venting, or adjunctive devices, such as an intra-aortic balloon pump or Impella, to decompress the ventricle or other LV unloading techniques/procedures.^15^^,^^17^^,^^18^ One study described the use of a bifurcated graft on the right axillary artery to accommodate both a VA-ECMO cannula and an Impella device for simultaneous LV unloading. Other studies did not provide sufficient detail on Impella access sites.

The impact of left-sided VA-ECMO flow from the left axillary or subclavian artery access appear less dangerous from a flow dynamics standpoint, more likely in proximity with the descending aorta and therefore generating a more antegrade flow, but likely less coronary or carotid perfusion. These flow dynamics aspects have been neglected so far, however, and warrant specific investigations, which are currently ongoing by our group.

### Complications and Outcomes

The studies reported various complications associated with subclavian or axillary artery cannulation in VA-ECMO patients. No cases of arterial dissection related to direct cannulation were explicitly documented in the reviewed studies; nonetheless, this complication remains theoretically possible, particularly with repeated cannulations or in patients with fragile vessels, and should be monitored for in clinical practice. The inability to complete cannulation—whether due to anatomic, technical, or procedural challenges—was not reported consistently. As a result, the overall feasibility and success rate of axillary or subclavian artery cannulation could not be assessed definitively. Some studies described conversion from femoral to subclavian/axillary cannulation to address such complications as limb ischemia or to enable prolonged support and patient mobilization. However, the number of such cases was not systematically reported, limiting quantitative analysis. Bleeding was a significant concern, with the highest rate reaching 17.3%, emphasizing the need for meticulous surgical techniques and appropriate anticoagulation management as well as monitoring. The reported rate of limb ischemia was as low as 1.2% in some studies, while other studies documented rates of up to 9.6%. This wide range likely reflects differences in patient selection, use of limb protection strategies, and reporting rigor and should be interpreted with caution when compared to femoral access–related ischemia rates.

Some centers advocate the use of a distal perfusion catheter even with upper limb–related arterial cannulation.^5^^,^^13^^,^^18^^,^^24^ Although graft-based cannulation theoretically preserves distal perfusion, some centers applied distal perfusion catheters selectively in patients with high flow demands, peripheral vascular disease, or clinical evidence of hypoperfusion to ensure adequate distal limb perfusion. Overall infection rates were reported (Table 4); none of the included studies explicitly distinguished between superficial wound infections and graft-related infections. As a result, the true incidence of graft-specific infectious complications remains unclear and likely underreported. Other complications include accidental cannula dislodgement,^15^ kinking, or malpositioning, which can impair flow or cause massive bleeding.^32^ None of the included studies specifically reported hemolysis rates or biochemical markers (eg, lactate dehydrogenase, free hemoglobin) in relation to the cannulation technique. This represents a relevant but underexplored complication that may be associated with cannula malposition, high flow rates, or shear stress at the graft–vessel interface.

During decannulation, there is a risk of bleeding and pseudoaneurysm formation owing to improper closure of the arterial puncture site.^6^ Reported infection rates were notably low or even absent in some studies, such as in the cohort study reported by Chamogeorgakis and colleagues.^4^ Hyperperfusion syndrome was a more prevalent complication, however, affecting up to 24.7% of patients, particularly those with graft-based cannulation. This condition can lead to localized damage in the limb supplied by the cannulated artery, underlining the importance of careful flow modulation and hemodynamic monitoring. Data on explantation strategies for graft-based cannulation—whether complete graft removal, partial resection, or distal ligation—were rarely reported. This lack of detail prevents a thorough evaluation of post-decannulation complications, such as graft thrombosis, infection, and late pseudoaneurysm formation.

Brachial plexus injury, although relatively rare, was reported at a rate of 2.5%.^4^ This complication may result from mechanical compression or direct trauma during the cannulation procedure and highlights the importance of precise surgical placement to minimize nerve injury. Additionally, reported rates of pulmonary edema ranged from 1.3% to 6.3%.^4^^,^^15^^,^^18^^,^^21^ This may be linked to challenges in LV unloading, which were not addressed uniformly in the reviewed studies. Stroke risk appears to be a major limitation of axillary/subclavian cannulation, with multiple comparative studies indicating higher cerebrovascular event rates compared to femoral access (Table 4). Across studies that provided this information, stroke rates ranged from 5.2%^18^ to 18.8%.^22^ Stroke rates stratified by extracorporeal pulmonary resuscitation (eCPR) status or prior cardiac arrest were not reported systematically in the included studies. A recent report by Liu and colleagues^33^ described successful axillary artery cannulation in 3 eCPR patients, with no cranial infarcts observed on imaging post–VA-ECMO. Our group is also preparing an analysis of the Extracorporeal Life Support Organization registry to investigate neurologic complications in eCPR patients undergoing axillary artery cannulation. While this risk may reflect in part patient comorbidity or hemodynamic complexity, further investigation is warranted to determine whether flow dynamics or cannula positioning contribute to this elevated risk.

The dual-cannulation strategy (ie, combined axillary/femoral arteries) had comparable 30-day mortality rates (40.74% for femoral artery vs 33.33% for the combined cannulation group). The rate of complications such as limb ischemia was significantly lower in the combined group (8.33% vs 37.04%), showcasing its potential to mitigate vascular-based issues.

Complication rates stratified by etiology were reported inconsistently across studies, precluding robust subgroup analysis. Cross-referencing complication rates with predominant cohort etiologies might suggest trends but would risk overinterpretation. Future studies should address this gap to better inform risk stratification.

#### In-hospital and postdischarge outcomes

The duration of VA-ECMO support varied, with an average of 3 to 7 days in most studies. These findings indicate that subclavian or axillary artery cannulation techniques can provide effective support over a wide range of treatment periods, although complications, such as LV overload and pulmonary edema may influence weaning success. Pulmonary edema was noted in up to 6.3% of cases, indicating the need for better LV unloading strategies with this approach, which were reported inconsistently across the studies.^4^^,^^13^^,^^15^^,^^18^^,^^23^

Rates of successful VA-ECMO weaning ranged from 30.9% to 80%, with smaller studies^9^^,^^34^ reporting higher success rates, possibly owing to+ selection bias or differences in patient management. Data on transitions to advanced therapies, such as durable VAD implantation and heart transplantation, were not reported consistently across studies, precluding quantitative analysis. Future investigations should systematically capture these outcomes to evaluate the role of axillary/subclavian VA-ECMO as a bridge-to-bridge or bridge-to-transplant strategy. In-hospital or 30-day survival rates varied between 17.3% and 63.8%.^13^^,^^16^ Long-term survival data derived from the analyzed studies were sparse, precluding definitive conclusions about the sustained benefits of this cannulation approach; however, 1-year survival rates ranged from 25.7% to 50.6% (Table 4). Survival data stratified by etiology were not reported uniformly across studies, precluding robust subgroup comparisons. While cross-referencing survival rates with predominant cohort etiologies suggests potential trends, such an approach would risk overinterpretation. Future studies should provide detailed etiology-specific outcomes to guide patient selection and prognostication.

### Comparative Studies

Approximately one-third of patients experienced cannulation-related complications, with similar overall rates across axillary (or subclavian), femoral, and central approaches. Notably, axillary cannulation showed a lower incidence of limb ischemia compared to femoral access (Table E1). Neurologic events—including stroke and seizures—were more frequent with subclavian/axillary cannulation, with one large study reporting neurologic complications in nearly 20% of patients, compared to approximately 12% with femoral cannulation (Table E1). Moreover, aortic (central) cannulation was linked to higher in-hospital mortality, likely reflecting its use in sicker patients (particularly postcardiotomy) despite lower rates of neurologic complications, while peripheral (axillary and femoral) cannulation tended to result in more vascular complications overall, although survival rates were similar across methods. Additional findings indicated that axillary cannulation is associated with higher rates of hyperperfusion syndrome and bleeding complications, whereas femoral cannulation carries a higher risk of local infections, wound complications, and limb ischemia (Table E1).

## Clinical Implications and Future Directions

Complications such as stroke, bleeding, and hyperperfusion syndrome remain significant concerns in VA-ECMO with axillary/subclavian artery cannulation. Future research should focus on developing standardized protocols for anticoagulation, cannulation techniques, and monitoring to optimize outcomes. Studies with larger sample sizes and long-term follow-up also are needed to better understand the risk–benefit profile of this approach. Advances including minimally invasive techniques, enhanced monitoring tools, and the integration of LV unloading strategies hold promise for mitigating complications and expanding the use of axillary/subclavian VA-ECMO configuration. Patients supported with VA-ECMO in the context of eCPR were not systematically identified in the included studies. From the senior author's perspective, axillary artery cannulation during eCPR requires a specific skill set and choreography by a trained team to maintain sterility and high-quality chest compressions. Surgical axillary access is likely too time-consuming in this setting, while percutaneous ultrasound-guided access, although promising, has not yet been widely adopted. Liu and colleagues^33^ recently reported successful ultrasound-guided axillary artery cannulation for VA-ECMO in 3 eCPR patients, with spontaneous heartbeat recovery after conversion from femoral perfusion. This highlights a promising but underexplored area for future research.

### Limitations

This review has several limitations. First, the heterogeneity of the included studies, with varying sample sizes, clinical indications, and time periods, complicates direct comparisons of outcomes. Second, long-term follow-up data were limited, hindering assessment of the sustained impact of subclavian and axillary cannulation on survival and quality of life. In addition, variability in reporting complications, particularly neurologic events and LV unloading strategies or use, further limits the generalizability of the findings.

## Conclusions

Axillary or subclavian artery cannulation for VA-ECMO has emerged as a viable and increasingly used alternative to traditional femoral access, offering significant advantages such as antegrade flow with reduced afterload increase to the LV, improved cerebral perfusion, and reduced risk of limb ischemia. These benefits make it particularly suitable for selected patient populations, including those with contraindications to femoral cannulation or requiring prolonged VA-ECMO support. Its application is not without challenges, however, including technical complexity, stroke risk, and potential for hyperperfusion syndrome. Furthermore, while current evidence supports its efficacy, further dedicated studies carefully assessing hemodynamic as well as flow-related effects, particularly with a right-sided approach, are needed. With continued innovation and rigorous evaluation, subclavian and axillary cannulation is poised to play a critical role in advancing extracorporeal life support.

## Conflict of Interest Statement

Dr Lorusso reported receiving research support from Medtronic and LivaNova; consulting for Medtronic, Abiomed, and LivaNova, and serving on medical advisory boards for Eurosets, Hemocue, and Xenios. All other authors reported no conflicts of interest.

The *Journal* policy requires editors and reviewers to disclose conflicts of interest and to decline handling or reviewing manuscripts for which they may have a conflict of interest. The editors and reviewers of this article have no conflicts of interest.

## Figures and Tables

**Table E1:** Key findings of studies comparing complications and outcomes of different cannulation sites for VA-ECMO

Year	Author	Patients (n)	Cannulation configuration, n (%)	Key findings
2021	Ohira^14^	371	Axillary: 218 (58.7); femoral: 153 (41.2)	Leg ischemia higher in femoral (15.7%) than axillary (6.9%) Wound complications higher in femoral (15%) vs axillary (2.8%) Similar survival rates (axillary, 60.6%; femoral, 56.9%) Axillary safer in patients with peripheral vascular disease
2022	Moussa^15^	372	Subclavian: 52 (14.0); femoral: 320 (86.0)	Subclavian did not reduce LV distention-related complications Increased serious bleeding and accidental decannulations with subclavian No difference in 28-d mortality
2022	Schaefer^16^	436	Axillary: 250 (57.3); femoral: 186 (42.6)	Limb ischemia higher in femoral (16.7%) than axillary (4.8%) Stroke incidence higher in axillary (11.2%) vs femoral (2.2%) No survival difference at 30 d or 1 y
2024	Do Vale^18^	333	Axillary: 209 (62.8); femoral: 124 (37.2)	90-d mortality similar in the 2 groups (54% vs 58%) Axillary cannulation had: Lower rates of limb ischemia (OR, 0.37) Lower rates of bowel ischemia (OR, 0.16) Lower rate of local infections (OR, 0.21) Higher stroke rate (OR, 2.87)
2013	Chamogeorgakis^4^	308	Aortic: 61 (19.8); axillary/subclavian: 81 (26.3); femoral: 166 (53.9)	Axillary complications: hyperperfusion (24.7%), bleeding (17.3%) Femoral complications: limb ischemia (16%), fasciotomy (10.8%) Cannulation type not a predictor of in-hospital mortality
2017	Ranney^22^	131	Aortic: 36 (27.5); axillary/subclavian: 16 (12.2); femoral: 79 (60.3)	Peripheral cannulation (axillary/femoral) had higher vascular complications (29.5%) vs ventral cannulation (mediastinal bleeding, 11.1%) Stroke rates and survival similar across all strategies
2017	Wong^11^	103	Aortic: 26 (25.0); axillary/subclavian: 41 (40.0); femoral: 36 (35)	Cannulation-related complications: 32% overall (axillary, 34%; femoral, 36%; central, 23%) Hyperperfusion: axillary, 15% (*P* = .01) Limb ischemia: femoral, 25% (*P* < .01) No difference in mortality between groups
2024	Chiarini^17^	1897	Aortic: 503 (26.5); axillary/subclavian: 397 (20.9); femoral: 997 (52.6)	Neurologic complications (stroke, hemorrhage, brain edema): axillary/subclavian, 19.6%; aortic, 15.8%; femoral, 11.9% (*P* < .001) Seizures: axillary/subclavian, 3.4%; aortic, 1.8%; femoral, 1.3% (*P* = .036) Mortality: aortic, 68.4%; femoral, 58.9%; subclavian/axillary, 56.2%

*LV*, Left ventricular; *OR*, odds ratio.

**Table E2:** Key findings on indications for subclavian/axillary artery cannulation for VA-ECMO

Indication	Description	Rationale	References
Peripheral vascular disease	Patients with severe arterial disease in the femoral or iliac vessels	Axillary artery has minimal atherosclerotic involvement compared to femoral vessels	4, 9
Groin infections or poor wound healing	Presence of active infection or poor tissue quality in the femoral region	Reduces the risk of infection and wound complications associated with femoral access	4, 15
Need for antegrade flow	Cases where cerebral or coronary perfusion is compromised (eg, differential hypoxia)	Flow from axillary cannulation ensures better perfusion to the brain and heart	4, 17
Prolonged VA-ECMO support	Patients requiring long-term mechanical circulatory support	Facilitates patient mobility and reduces complications like limb ischemia	14, 16
Retrograde flow complications	Patients with LV overload, pulmonary edema, or thromboembolic risks	Axillary cannulation avoids retrograde flow, reducing LV afterload and associated complications	16
Limited groin access	Prior femoral interventions, stents, or vascular surgeries	Axillary artery provides an alternative site when femoral access is not feasible	4, 15
High risk for limb ischemia	Cases with previous limb ischemia or high ischemic risk with femoral cannulation	Axillary cannulation has a significantly lower incidence of limb ischemia	17, 19
Proximity to thoracic procedures	Patients undergoing concomitant thoracic or cardiac surgery (eg, aortic dissection repair)	Axillary artery is accessible during surgical exposure	5, 9

*VA-ECMO*, Venoarterial extracorporeal membrane oxygenation; *LV*, left ventricular.

**Table E3:** Key findings on cannulation techniques for subclavian/axillary artery VA-ECMO

Technique	Description	Advantages	Challenges	References
Direct cannulation	Cannula is inserted directly into the axillary artery without a graft	Faster setup Reduces hyperperfusion syndrome Simplifies procedure	Increased risk of arterial injury Potential for bleeding	4, 9, 16
Graft interposition technique (cannula tip in artery)	A Dacron or PTFE graft is sewn to the axillary artery, and the cannula is inserted into the artery	Reduces arterial trauma Facilitates flow control Allows better cannula stability	Longer surgical time Increased bleeding risk at the graft site Risk of infection	4, 9, 17
Graft interposition technique (cannula tip in graft)	A Dacron or PTFE graft is sewn to the axillary artery, and the cannula is inserted into the graft with tip position proximal to the anastomosis	Maintains native arterial integrity Reduces risk of limb ischemia	Longer surgical time Increased bleeding risk at the graft site Risk of infection Risk of hyperperfusion	16
Percutaneous axillary cannulation	Cannula is inserted using a percutaneous technique guided by ultrasound or fluoroscopy	Minimally invasive Shorter procedure time Suitable for emergencies	Limited availability of expertise Risk of arterial puncture complications Increased risk on removal of cannula	15, 19
Bifurcated graft	Combined use of VA-ECMO with Impella for LV unloading	Flexible approach Single access for 2 interventions	Complex planning needed Not widely practiced or standardized Risk of bleeding and infection	21

*PTFE*, Polytetrafluoroethylene; *VA-ECMO*, venoarterial extracorporeal membrane oxygenation; *LV*, left ventricular.

**Table E4:** Search strategy

Database	Strategy
PubMed	(“Extracorporeal Membrane Oxygenation” [Mesh] OR “Extracorporeal Circulation” [Mesh] OR “Oxygenators, Membrane” [Mesh] OR “membrane oxygenator∗” [All Fields] OR “extracorporeal circulation∗” [All Fields] OR “extracorporeal life support∗” [All Fields] OR “extracorporeal membrane oxygenation∗” [All Fields] OR ECMO [All Fields] OR ECLS [All Fields]) AND (cannulation∗[All Fields] OR “Catheterization” [Mesh] OR catheteri∗ation∗[All Fields] OR “arter∗ access” [All Fields]) AND (“Subclavian Artery” [Mesh] OR “Axillary Artery” [Mesh] OR “Brachiocephalic Trunk” [Mesh] OR “subclavia∗ arter∗” [All Fields] OR “axillar∗ arter∗” [All Fields] OR “axillar∗ trun∗” [All Fields] OR “subclavia∗ trun∗” [All Fields] OR “anonym∗ arter∗” [All Fields] OR “anonym trun∗” [All Fields ] OR “brachiocephalic∗ arter∗” [All Fields] OR “brachiocephalic trun∗” [All Fields] OR “innominat∗ trun∗” [All Fields] OR “innominat∗ arter∗” [All Fields] OR “upper limb arter∗” [All Fields])
Web of Science	ALL=(“membrane oxygenator∗” OR “extracorporeal circulation∗” OR “extracorporeal life support∗” OR “extracorporeal membrane oxygenation∗” OR ECMO OR ECLS) AND ALL=(cannulation∗ OR catheteri?ation∗ OR “arter∗ access”) AND ALL=(“subclavia∗ arter∗” OR “axillar∗ arter∗” OR “axillar∗ trun∗” OR “sublcavia∗ trun∗” OR “anonym∗ arter∗” OR “anonym trun∗” OR “brachiocephalic∗ arter∗” OR “brachiocephalic trun∗” OR “innominat∗ trun∗” OR “innominat∗ arter∗” OR “upper limb arter∗”)
Embase	(‘extracorporeal oxygenation/exp OR ‘extracorporeal circulation'/exp OR ‘membrane oxygenator’/exp OR ‘membrane oxygenator∗’ OR ‘extracorporeal circulation∗’ OR ‘extracorporeal life support∗’ OR ‘extracorporeal membrane oxygenation∗' OR ‘ecmo’ OR ‘ecls’) AND (‘cannulation∗’ OR ‘artery catheterization’/exp OR ‘catheteri?ation∗’ OR ‘arter∗ access’) AND (‘subclavian artery’/exp OR ‘axillary artery'/exp OR ‘brachiocephalic trunk’/exp OR ‘subclavia∗ arter∗’ OR ‘axillar∗ arter∗' OR ‘axillar∗ trun∗’ OR ‘subclavia∗ trun∗’ OR ‘anonym∗ arter∗' OR ‘anonym trun∗’ OR ‘brachiocephalic∗ arter∗’ OR ‘brachiocephalic trun∗' OR ‘innominat∗ trun∗' OR ‘innominat∗ arter∗’ OR ‘upper limb arter∗’)
